# Risk factors for glial cell proliferation after idiopathic macular hole repair with internal limiting membrane flap

**DOI:** 10.1186/s12886-019-1265-0

**Published:** 2019-12-21

**Authors:** Yuyan Liu, Changlong Wu, Ying Wang, Yi Dong, Dongqing Liang, Bo Xiao, Quanhong Han, Yanhua Chu

**Affiliations:** 1 Tianjin Eye Hospital, Tianjin Key Laboratory of Ophthalmology and Visual Science, Tianjin Eye Institute, Clinical College of Ophthalmology, Tianjin Medical University, 4, Gansu Road, Heping District, Tianjin, 300020 China; 2 Jinan Second People’s Hospital, Ophthalmology, Jinan City, 250001 Shandong Province China

**Keywords:** Idiopathic macular hole, Internal limiting membrane flap, Glial cell proliferation, Vitrectomy, Optical coherence tomography

## Abstract

**Background:**

To study the influencing factors for different healing patterns of patients with idiopathic macular holes (IMH) after vitrectomy surgery performed with the internal limiting membrane (ILM) flap technique**.**

**Methods:**

This study was a retrospective, consecutive, observational case series study. We recruited 52 IMH patients who underwent vitrectomy with the ILM flap technique. The participants were divided into 2 groups: group A (25 patients), without significant glial cell proliferation in the macular area on postoperative optical coherence tomography (OCT); and group B (27 patients), with significant glial cell proliferation. The postoperative visual acuity (VA), external limiting membrane (ELM) and ellipsoid zone (EZ) recovery characteristics were compared between the two groups.

**Results:**

There were statistically significant differences in minimum linear diameter (MLD) of the macular hole and postoperative VA (*p* = 0.02, 2.81 E-4 respectively) between the two groups. Compared with patients in group A, patients in group B had poorer VA and EZ recovery in the first 12 months after surgery, and a longer ELM recovery period. The OCT results showed that patients in group B had more extensive ILM filling in the macular area after surgery than patients in group A.

**Conclusion:**

The presence of aberrant glial cell proliferation was related to a larger MLD of the IMH, and the filling approach for the ILM during the operation was related to the postoperative healing pattern and vision acuity.

## Background

Idiopathic macular hole is a common retinal disease that mostly affects elderly people [1]. In 1991, Kelly and Wendel first reported that vitrectomy is effective for the treatment of IMH [2]. The development of ILM peeling and ILM flap improved the closure rate of patients with macular hole [3]. Compared with patients without ILM flaps, patients with ILM flaps have improved postoperative VA [4]. Recently, vitrectomy with ILM peeling or flaps has resulted in successful hole closure in 90–100% of IMH patients and visual improvement in over 85% of cases [5, 6]. However, postoperative visual acuity is unpredictable and occasionally unsatisfactory in some patients with anatomic closure. Published reports demonstrated that the postoperative status of the ELM and EZ layer significantly correlates with the VA outcome in IMH patients after surgery [1, 7–9], and is negatively related to aberrant glial cell proliferation [10, 11]. Recently, developed Spectral domain OCT (SD-OCT) with 5 μm high resolution has allowed observation of ELM and the ellipsoid zone (EZ) to be more precise.

The purposes of this study were to investigate the long-term restoration changes of the ELM and EZ layer and the correlation of these structures with visual recovery after successful IMH vitrectomy and ILM flap surgery, and to determine the possible factors that may be associated with glial cell proliferation in the macula retina.

## Methods

### Study design and participants

The study was a retrospective, consecutive, observational case series. The study followed the tenets of the Declaration of Helsinki and was approved by the Medical Ethics Committee of Tianjin Eye Hospital.

Patients who were diagnosed with IMH and had undergone 25G PPV and ILM flap surgery in Tianjin Eye Hospital between January 1, 2015 and May 31, 2017 were included. The exclusion criteria included prior vitreoretinal surgery, pathologic myopia [refractive error of more than − 6.00 diopters (D) or axial length (AL) more than 26.0 mm], neovascular age-related macular degeneration, proliferative diabetic retinopathy, solar retinopathy, and traumatic MH. Of the original 123 eyes, there were 65 eyes whose ILM could be found in the first time follow-up OCT images. A total of 5 eyes were excluded because of the presence of retinal diseases, including treated rhegmatogenous retinal detachment, diabetic retinopathy, and high myopia with an axial length of more than 26.0 mm or refractive error less than − 6.0 diopters. Eight other eyes were excluded because the patients were lost to follow-up within 6 months postoperatively. In total, 52 eyes of 52 patients (13 men, 39 women) met the study criteria for the data analysis. All the macular holes of 52 patients were closed after the first operation and ILM flap was attained surgically in these 52 eyes.

According to whether there was glial proliferation in the healing process of the macular hole one month post operation, the patients were divided into two groups. Patients with functional closure of the macular region were designated as group A, which is defined as the presence of normal layers in the macular region without blocking by hyper reflective substances. Patients with anatomical closure, which was characterized by discontinuous macular layers filled with hyper reflective material, were designated group B [11].

### Ophthalmologic examinations

**Fig. 1:**
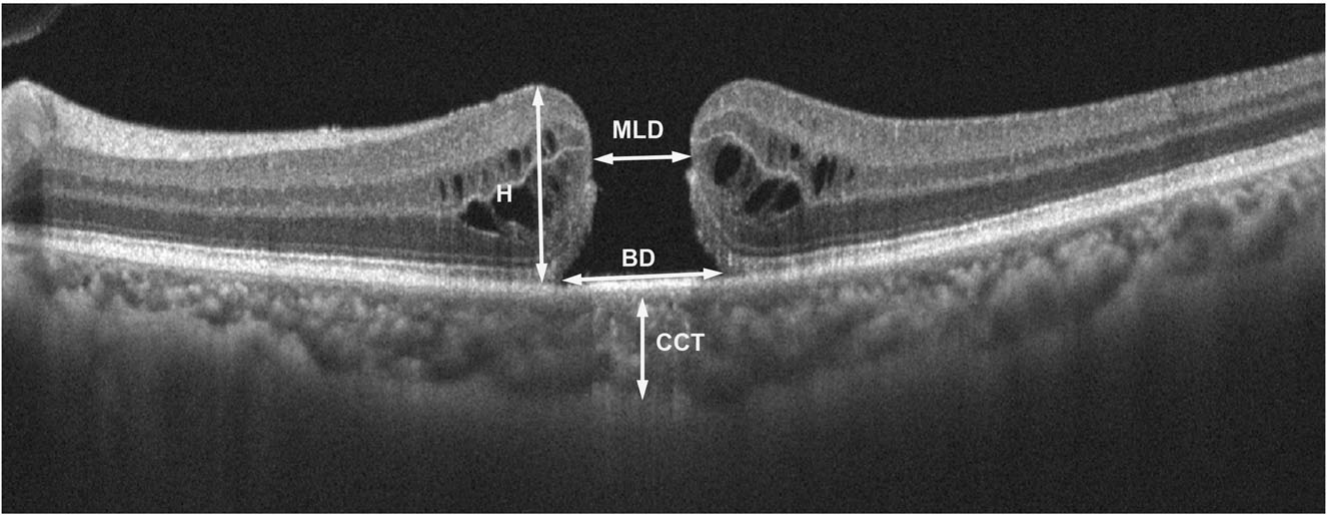
Examples of the preoperative measurement of the macular hole. This image is a horizontal scan of the OCT results through the macular fovea

A detailed eye examination including a slit-lamp examination, fundus examination by indirect binocular ophthalmoscopy, and SD-OCT (RTVue XR 100–2, Optovue, USA) to scan the foveal microstructures were performed. The preoperative data included age, sex, symptom duration, right or left eye, BCVA, AL, refractive status, MLD, height (H) and the base linear diameter (BD) of the macular hole, and the central choroid thickness (CCT). This SD-OCT device used an 840-nm wavelength, and a scanning speed of 70,000 A-scans/second. A 6 mm × 6 mm scanning pattern was performed. The MLD, BD and H of the macular hole and CCT were manually measured with the calipers included in the software (Fig. 1). Two masked professional physicians evaluated all the images with excellent inter-and-intra-observer reliability for all measured macular structures.

### Operation method

All the surgeries included in the study were performed by the same vitreoretinal specialist (H. QH.). A standard sutureless (25G) 3-port pars plana vitrectomy (PPV) was performed in all cases. An intravitreal injection of indocyanine green (5 mg/mL) was performed to make the ILM more visible. The ILM was peeled off in a circular fashion around the MH, and the remaining ILM around the macular hole was trimmed short then massaged gently over the MH until the ILM became inverted. Phacoemulsification with implantation of intraocular lens and circular dissection of the posterior capsule was performed simultaneously in patients with cataracts or those older than 50 years. Sterile air was used to tamponade the vitreous cavity in all patients, and patients were instructed to maintain a prone position while awake for at least 4 days postoperatively.

### Follow-up data

Comprehensive ophthalmologic examinations and SD-OCT examinations were performed 1, 3, 6, 9, and 12 months after surgery for all patients. The follow-up model of the SD-OCT device we used, which could identify previous scan locations and automatically guide the instrument to scan the same location again for every visit. The same scanning location was determined by the eye-tracking system but not by the location of the fixation light. In the process of image acquisition, the position of the fovea was manually repositioned for patients with eccentric fixation to correct the scanning deviation from the fovea. The examinations included BCVA, IOP, slit-lamp examinations, and fundus examinations by indirect binocular ophthalmoscopy. BCVA was converted to the logarithm of the minimum angle of resolution (logMAR) to evaluate VA changes and for statistical analysis. The length of the ELM and EZ line defect was analyzed by the horizontal B scan in the fovea region. These images were analyzed by two masked observers who evaluated all the photographs with inter and intra-observer reliability for all OCT data measured.

### Statistical analysis

Statistical analysis was carried out using SPSS, v 19.0 (IBM, Armonk, New York, USA). Continuous data were reported as the median ± standard deviation (SD). An independent sample *t*-test was used to compare data between groups. The chi-square test was used for categorical variables. A *p* < 0.05 was considered as statistically significant.

## Results

### Comparison of the preoperative data between group A and group B

Our study included 25 subjects in group A (median age: 65 years; 17 females, 8 males) and 27 subjects in group B (median age: 64 years; 22 females, 5 males). The mean and standard deviation of the preoperative data of the two groups and the statistical analysis results are shown in Table 1. The results of the comparison of preoperative basic data between group A and group B showed that only the difference in MLD was statistically significant, while the differences in age, symptom duration, AL, preoperative vision, refraction, BD and H of the macular hole and CCT between the two groups were not statistically significant. The difference in postoperative visual acuity was statistically significant between group A and group B, while the difference in visual acuity improvement was not statistically significant. The postoperative visual acuity in group A was better than that in group B, which may be related to the preoperative visual acuity and healing pathway.

**Table 1:** Basic data and comparative analysis results of groups A and B

Group	A(25)	B(27)	*t*	*P*
Age (year)	65.36 ± 4.94	63.92 ± 5.37	1.00	0.32
Pre-V	1.13 ± 0.40	1.25 ± 0.55	0.87	0.39
AL (mm)	23.38 ± 1.04	23.36 ± 1.04	0.08	0.94
D	−0.69 ± 1.81	−0.58 ± 2.73	− 0.17	0.84
BD (μm)	913.38 ± 311.00	1038.77 ± 280.26	−1.49	0.14
MLD (μm)	485.42 ± 173.66	604.69 ± 159.72	−2.53	0.02
H (μm)	407.21 ± 89.00	407.50 ± 77.13	−0.01	0.99
CCT (μm)	201.29 ± 61.17	224.85 ± 74.42	−1.22	0.23
Duration (month)	5.63 ± 9.56	5.96 ± 4.30	−1.65	0.87
Post-V	0.24 ± 0.16	0.51 ± 0.31	3.40	2.81E-4
Increase-V	0.90 ± 0.38	0.74 ± 0.44	1.34	0.19

### Comparison of the postoperative VA between group A and group B

**Fig. 2:**
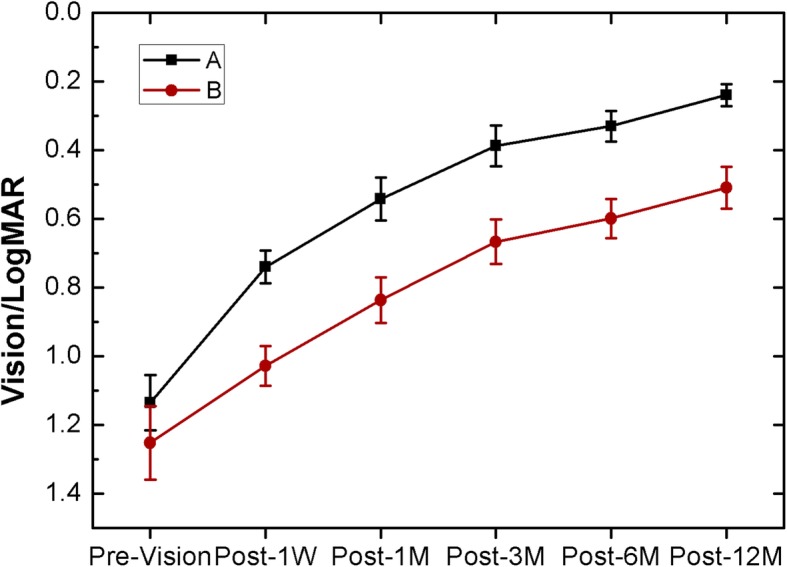
Preoperative and postoperative visual acuity changes of the two groups at different times

Figure 2 shows the preoperative VA of group A at 1 week, and 1, 3, 6 and 12 months post operation. The BCVA improved from 1.14 ± 0.08 preoperatively to 0.74 ± 0.05 at 1 week, 0.54 ± 0.06 at 1 month, 0.39 ± 0.06 at 3 months, 0.33 ± 0.04 at 6 months, and 0.23 ± 0.03 at 12 months postoperatively in group A**.** The BCVA in Group B improved from 1.25 ± 0.11 preoperatively to 1.03 ± 0.06, 0.84 ± 0.07, 0.67 ± 0.07, 0.60 ± 0.06 and 0.51 ± 0.06 at 1 week, 1, 3, 6, and 12 months postoperative respectively**.** There were no significant differences in the preoperative VA between groups A and B, while the differences in postoperative VA were statistically significant at different follow-up times after surgery (the *p* value at 1, 3, 6 and 12 months were 3.06E-3, 2.49 E-3, 5.37E-4, and 2.89E-4, respectively). The postoperative visual acuity of groups A and B gradually increased, and the postoperative visual acuity of group B was lower than that of group A.

### Comparison of the changes in the ELM and EZ characteristics in the macular area between two groups at different times post operation

**Fig. 3:**
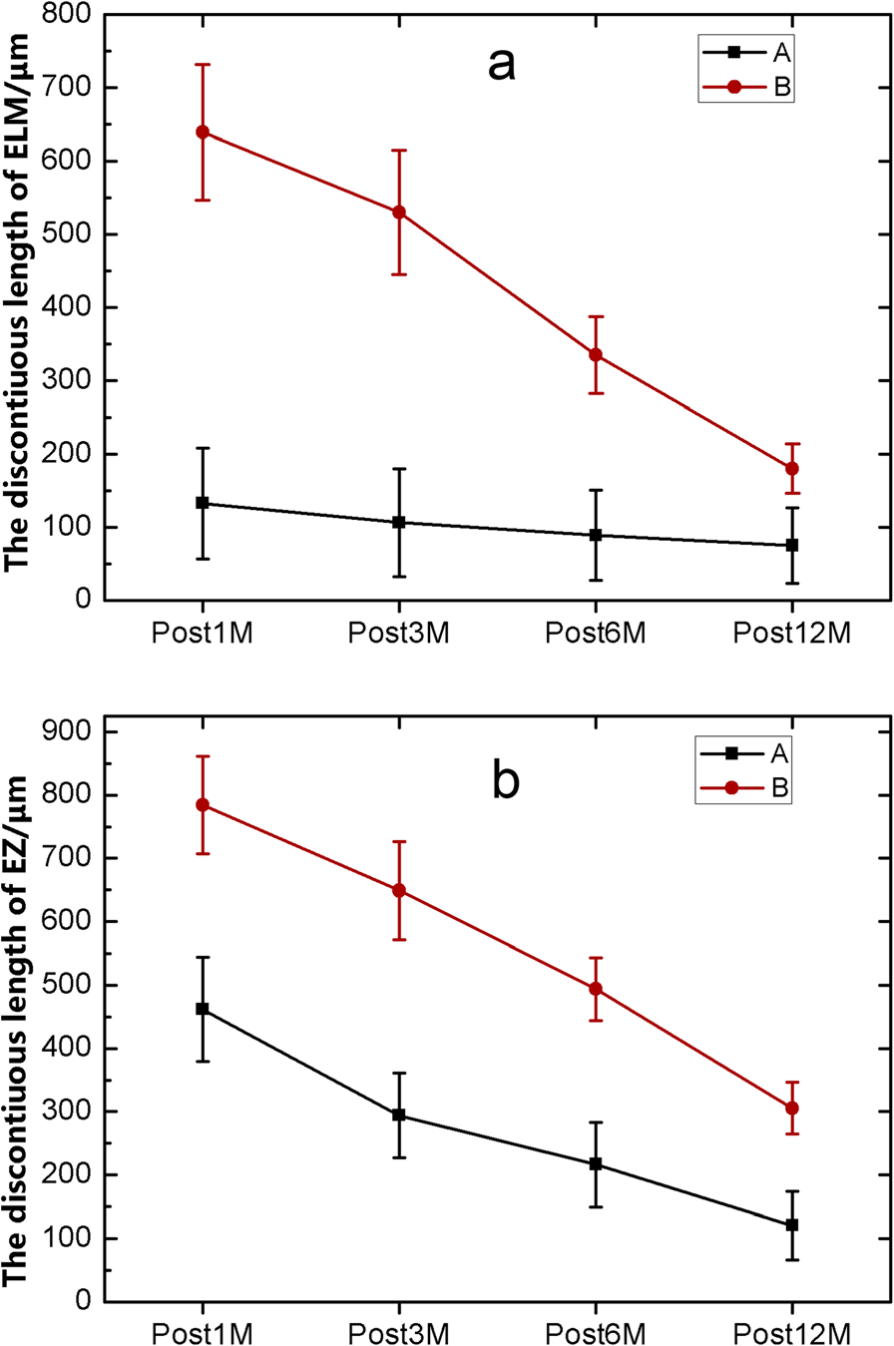
The variation trend of the lengths of the ELM (**a**) and EZ (**b**) discontinuity in the macular area in the two groups at different times post operation

The changes in the characteristics of the length of ELM defect post operation in groups A and B are shown in Fig. 3a. The length of the ELM discontinuity in group A gradually decreased at 1, 3, 6 and 12 months after surgery, and the length of the ELM defect in group B was slowly restored in the corresponding times post operation. A comparison between group A and group B showed that the differences of the length of the ELM discontinuity at 1, 3 and 6 months postoperatively were statistically significant (*p* = 9.41E-5, 4.14E-4, and 5.37E-4, respectively), while the difference in the length of ELM defect at 12 months post operation was not statistically significant (*p* = 0.09) (Table 2). The length of the EZ discontinuity at different times after surgery in the groups A and B are shown in Fig. 3b. The length of the EZ discontinuity in group A and group B became gradually restored at 1, 3, 6 and 12 months post operation. A comparison between group A and group B showed that the differences in the length of the EZ defect at 1, 3, 6 and 12 months after surgery was statistically significant (*p* = 5.74 E-3, 1.03E-3, 1.36E-3, and 7.84E-3, respectively) (Table 2).

**Table 2:** The data and statistical results of postoperative visual acuity, the discontinuity length of ELM and EZ in the two groups

Group	A(25)	B(27)	t	*P*
Post 1 M				
Vsion	0.54 ± 0.31	0.82 ± 0.34	3.11	3.06E-3
ELM (μm)	132.48 ± 378.53	639.33 ± 472.58	−4.25	9.41E-5
EZ (μm)	461.48 ± 412.86	784.15 ± 393.18	−2.89	5.74 E-3
Post 3 M				
Vsion	0.39 ± 0.30	0.67 ± 0.33	3.12	2.49 E-3
ELM (μm)	106.00 ± 367.36	530.15 ± 434.89	−3.78	4.14E-4
EZ (μm)	294.16 ± 334.40	648.78 ± 393.94	−3.49	1.03E-3
Post 6 M				
Vsion	0.33 ± 0.22	0.59 ± 0.29	3.70	3.34E-3
ELM (μm)	88.80 ± 307.36	335.00 ± 268.57	−3.08	5.37E-4
EZ (μm)	216.36 ± 334.49	493.89 ± 252.25	−3.39	1.36E-3
Post 12 M				
Vsion	0.24 ± 0.16	0.51 ± 0.31	3.90	2.89E-4
ELM (μm)	74.80 ± 259.22	493.89 ± 252.45	−1.74	0.09
EZ (μm)	120.40 ± 270.46	305.41 ± 209.32	−2.77	7.84E-3

### Comparison of the microstructures of the macular area post operation between the two different healing methods

**Fig. 4:**
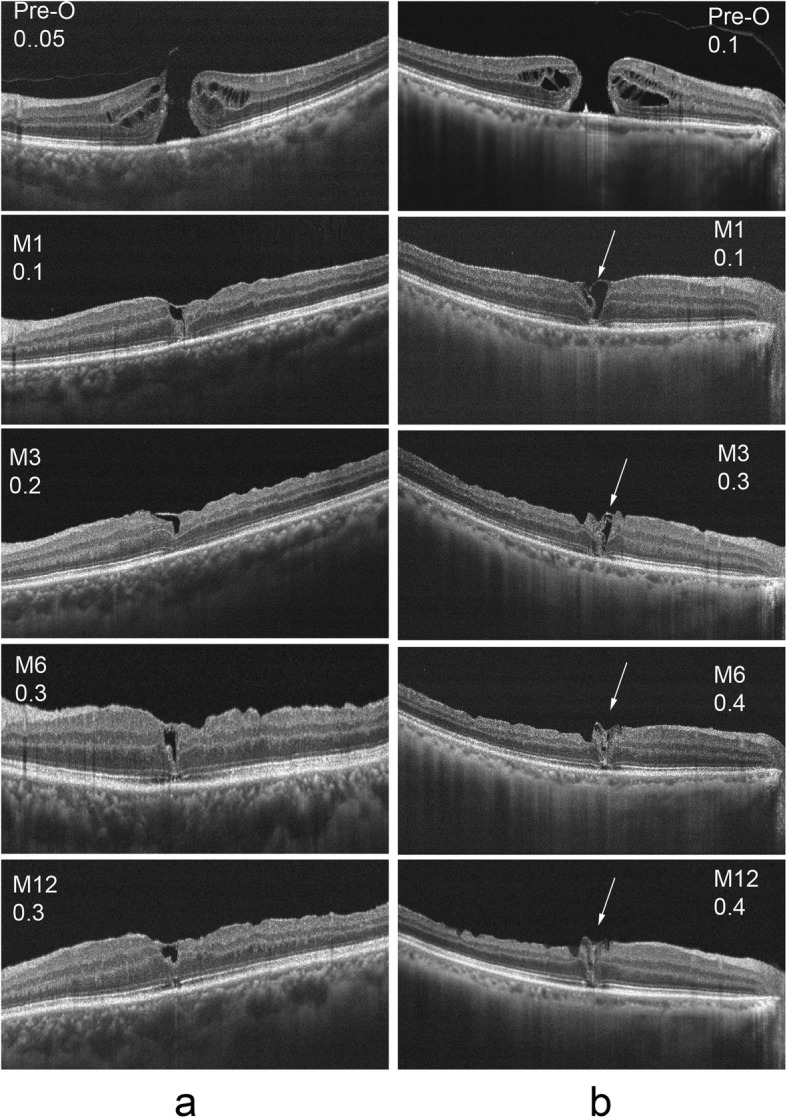
Visual acuity and OCT results pre and post operation of a patient from group A were shown in column **a**, and the results of a patient from group B were shown in column **b**. The proliferation of glial cells were shown with arrows in column **b**

**Fig. 5:**
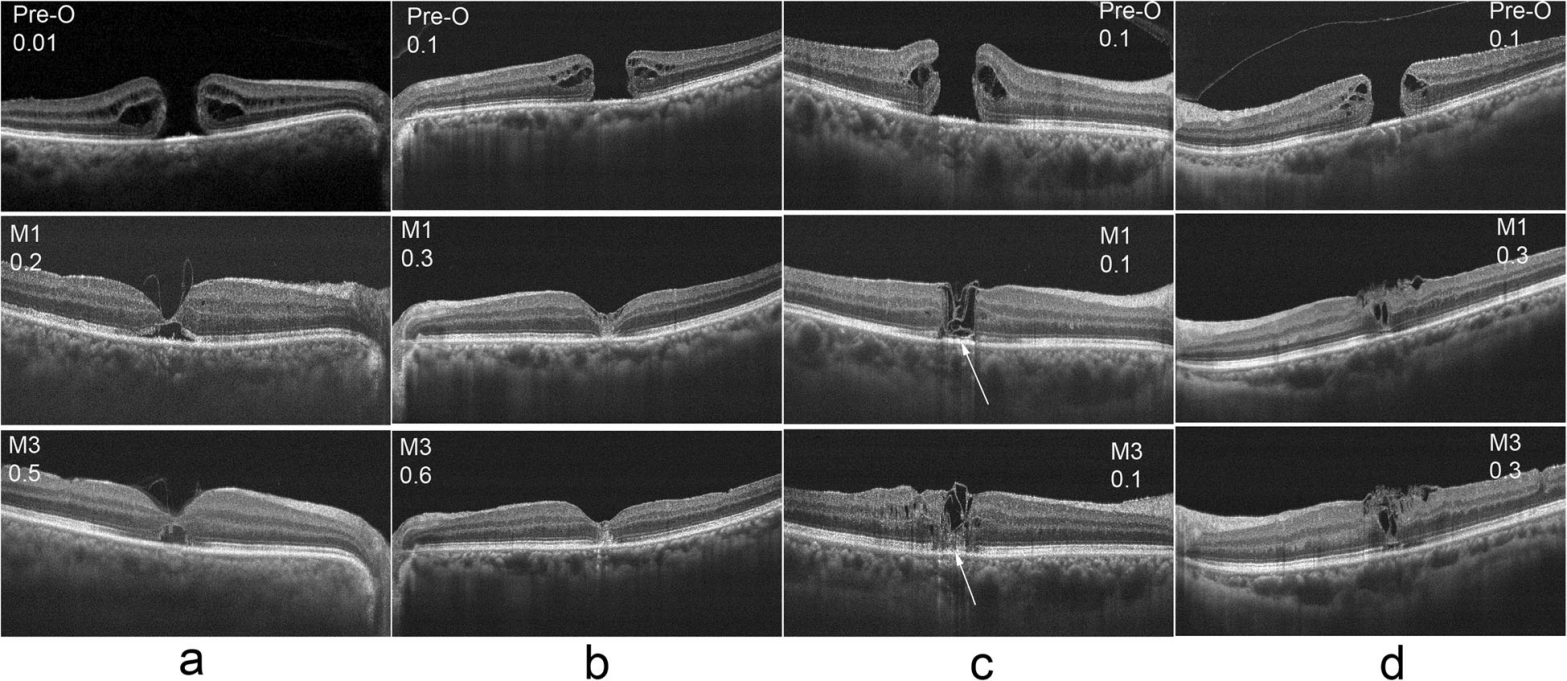
Visual acuity and OCT results before and after operation were observed in the two groups. The results of a patient from group A were shown in column **a**, and the results of three patients from group b were shown in column **b**, column **c** and column **d**, respectively. The patient of column **c** had a deeper filling depth (shown with arrows) of the ILM during the operation than which of the patient of column **d**

In addition to analyzing the differences between the two groups in postoperative VA, and ELM and EZ of the macular area, analyzing the OCT characteristics of the macular area postoperatively of specific patients might illustrate the differences between the two groups. As shown in Figs. 4 and 5, patients in group A had a rapid recovery of the ELM post operation and recovered 1 month after surgery, while patients in group B needed a longer time for ELM recovery. During the entire observation period after surgery, EZ recovery in both groups was slow. However, it can be seen from Fig. 5 that even if there was no intact ELM and EZ postoperatively, as long as the defect length was small, the patient had a chance to achieve good VA, which might be related to the development of partial fovea fixation in patients after surgery. In addition, through an analysis of the OCT data shown in Fig. 5, it can be seen that patients in group B had more ILM filling in the early postoperative stage and that the filling occupied the whole macular hole area, while patients in group A had less ILM filling in the early postoperative stage and the filled ILM was located in the shallow layer of macular hole area in the form of bridges. Longer ELM and EZ defect lengths and more glial cell proliferation were associated with relatively poorer postoperative VA.

## Discussion

### The relationship between preoperative data and glial cell proliferation

The preoperative data showed that only the difference in MLD between the two groups of macular holes was statistically significant. Patients with larger MLDs of the macular hole have a greater chance of developing the second healing pattern, which is similar to the results reported in 2017 about the microstructure of the macula area after MH surgery with the ILM flap technique [12].

In this study, patients with aberrant active glial proliferation in the macular area had poor VA. Glial proliferation may impact photoreceptor reconstruction, and the study showed that glial proliferation may occur when the foveal photoreceptors are impaired, which may be related to a greater hole diameter and longer hole duration [11]. Our results showed that only the minimum diameter of the hole but not the symptom duration may be related to glial hyperplasia, which may be related to the course duration since the patients in our study mostly had symptom duration of less than 2 years. However, we cannot exclude MLD as a confounding factor between the two groups that might influence the results.

### Relationship between the outer microstructure of the macular area and glial cell proliferation post operation

Lengths of the ELM and EZ discontinuities have been correlated with foveal sensitivity at 6 months postoperatively [13]. Statistical analysis of the data at 6 months post operation from the two groups showed that the differences between the length of the ELM and EZ defects in the macular area were statistically significant, and the postoperative microstructure in the macular area of group A was better than that of group B. A complete EZ was always accompanied by an intact ELM, whereas the opposite was not observed [11], which is consistent with our study.

Botti et al. showed that the ELM was the first layer to recover after macular hole surgery, followed by a gradual restoration of the EZ integrity [14]. An intact ELM has been considered to be a sign of intact photoreceptor cells and Müller cells, and the EZ junction rarely recovers without recovery of the ELM [13–16]. The integrity of the ELM, which is the junction between the inner segment and the Müller cells, was another important factor for the survival of photoreceptor cells and played a critical role in visual recovery [15, 17].

The postoperative visual acuity of the patients in group B was worse than that of the patients in group A within 1 year post operation, and the ELM and EZ recovery was slower in group B than that in group A, indicating that the recovery of VA and microstructures in the macular area was slower in group B than in group A, the ELM and EZ layer gradually improved after surgery, and visual functions steadily improved over time [18, 19]. This finding indicates that the complete recovery process of the macular microstructure and visual function may take at least 1 year [20, 21].

Recent in vivo studies suggest that glial tissues are essential for MH healing. However, severe gliosis may indicate a worse visual prognosis, and the disappearance of glial proliferation in the early postoperative period predicted better visual recovery [20]. The study that observed the early process of macular hole healing showed that the ELM was the first layer to recover after surgery, and some researchers considered that the newly formed ELM might prevent the growth of glial tissues.

### Effects of surgical procedures on glial cell proliferation

This study is of great importance in influencing the approach of ILM handling for future IMH surgeries. With the purpose to achieve ideal postoperative VA, we can make MH healing following the healing pattern of group A, the ILM flap was superior like a bridge rather than filling as tamponade during the operation. Park and colleagues described this phenomenon in a recent article: the ILM covered the top of the hole or plugged the hole [22]. Through observation, we found that plugging the ILM in outer layer of the retina promoted the proliferation of glial cells, prolonged the recovery time of normal tissue structures and even had a negative effect on the recovery of vision. More glial cell proliferation occurred in group B than in group A, which may be due to the effect of more extensive ILM filling during the surgical process for group B.

## Conclusion

Postoperative VA is unpredictable and occasionally unsatisfactory in some IMH patients with anatomic closure. The presence of aberrant glial cell proliferation was related to a larger MLD of the IMH, and the filling approach for the ILM during the operation was related to the postoperative healing pattern and VA.

## Data Availability

The data used in the current study is available from the corresponding author upon request.

## Abbreviations

AL: Axial length
BD: Base linear diameter
CCT: Central choroid thickness
D: Diopters
ELM: External limiting membrane
EZ: Ellipsoid zone
ILM: Internal limiting membrane
IMH: Idiopathic macular holes
logMAR: Minimum angle of resolution
MLD: Minimum linear diameter
OCT: Optical coherence tomography
PPV: Pars plana vitrectomy
SD-OCT: Spectral domain OCT
